# Nei Endonuclease VIII-Like1 (NEIL1) Inhibits Apoptosis of Human Colorectal Cancer Cells

**DOI:** 10.1155/2020/5053975

**Published:** 2020-06-26

**Authors:** Wanjuan Xue, Yongcheng Liu, Ningning Xin, Jiyu Miao, Juan Du, Yu Wang, Haiyan Shi, Yameng Wei, Huahua Zhang, Yani Chen, Yi Gao, Dan Li, Yun Feng, Jing Yan, Jing Zhang, Ni Hou, Chen Huang, Jiming Han

**Affiliations:** ^1^Department of Cell Biology and Genetics, Medical College of Yan'an University, Yan'an, 716000 Shannxi, China; ^2^Department of Pathology, Yan'an University Affiliated Hospital, Yan'an, 716000 Shannxi, China; ^3^School Infirmary, XianYang Normal University, Xianyang, 712000 Shannxi, China; ^4^Department of Cell Biology and Genetics, School of Basic Medical Sciences, Xi'an Jiaotong University Health Science Center, Xi'an, 710061 Shannxi, China

## Abstract

The study is aimed at investigating the role of Nei endonuclease VIII-like1 (NEIL1) in the pathogenesis of colorectal cancer (CRC). The human CRC (HCT116 and SW480) cells were subjected to the siRNA silencing and recombinant plasmid overexpression of NEIL1. Transfection of siNEIL1 significantly inhibited the cell growth. It also increased the Bax expression levels, while it decreased the Bcl-2 expression levels in human CRC cells, leading the Bax/Bcl-2 balance toward apoptosis. Moreover, the apoptosis was promoted through the caspase-9 signaling pathway. One the other hand, high expression of NEIL1 promoted the cell viability and reduced the apoptosis, inducing the balance of Bax/Bcl-2 in the human colon cancer cells to be antiapoptotic. In addition, the caspase-9 signaling pathway inhibited apoptosis, contrary to the results obtained by downregulating NEIL1 expression. Furthermore, NEIL1 was negatively regulated by miR-7-5p, indicating that miR-7-5p inhibited the NEIL1 expression after transcription. Overexpression of miR-7-5p reversed the effects of NEIL1 on these CRC cells. In conclusion, NEIL1 promotes the proliferation of CRC cells, which is regulated negatively by miR-7-5p. These findings suggest that NEIL1 is a potential therapeutic target for CRC.

## 1. Introduction

Occurrence and progression of colorectal cancer (CRC) might be associated with the accumulation of mutations of tumor suppressor genes and oncogenes [1]. Defects in the DNA damage repairing systems could lead to increased gene mutation rates and promote tumorigenesis and progression. BER is an important means of DNA damage repair mechanism, which plays an important role in removing the DNA base damage, maintaining the genomic stability, and preventing cancer pathogenesis. Nei endonuclease VIII-like1 (NEIL1) is a DNA repairing enzyme belonging to a class of DNA glycosylation enzymes homologous to the Fpg/Nei bacterium family, which are mainly involved in the mammalian base excision [2]. The *NEIL1* gene polymorphism is closely related to tumorigenesis [3]. The G83D mutation of the *NEIL1* gene can induce genomic instability and cell transformation [4]. The inactivating mutation of *NEIL1* disrupts the DNA repairing system, and the accumulation of bases damaged by oxidative stress would lead to the development of gastric cancer [5]. *NEIL1* is an essential and a ubiquitously edited ADAR1 target in multiple myeloma [6]. In CRC, *NEIL1* has abnormally high methylation levels [7]. The *NEIL1* IVS1 mutation could promote the susceptibility to CRC [8]. However, the role of *NEIL1* in the progression of CRC and the specific regulating mechanisms has rarely been elucidated.

MicroRNAs (miRNAs) can negatively regulate the gene expression after transcription by binding to the 3′-untranslated region (3′-UTR) of the target gene [9]. It has been shown that miRNAs are closely related to various biological processes, including cell proliferation, differentiation, apoptosis, and tissue development, which might also be involved in the occurrence and development of human cancers. miRNA- (miR-) 7 is an evolutionarily conserved miRNA abundantly expressed in the human pancreas and endocrine cells, which plays specific roles in the endocrine cell differentiation and function [10]. Moreover, it has been shown that miR-7 is associated with the progression of various tumors, including gastric cancer, lung cancer, breast cancer, and glioma [11]. DNA methylation-mediated miR-7-5p silencing would promote the gastric cancer stem cell invasion by increasing Smo and Hes1 [12]. Furthermore, methylation of miR-7 can be used as a biomarker for predicting the poor survival in patients with non-small cell lung cancer at the early stage.

In this study, the role of NEIL1 in the pathogenesis of CRC was investigated. The human CRC cells were subjected to the siRNA silencing and recombinant plasmid overexpression of NEIL1. Cell proliferation and apoptosis were detected. Moreover, the target-regulating miRNAs for NEIL1 were also predicted and confirmed.

## 2. Materials and Methods

### 2.1. Cell Culture

Human CRC cell lines (i.e., the HCT116 and SW480) and the normal human renal epithelial cell line (i.e., the HEK293) were obtained from the Key Laboratory of the Environmental and Disease Related Genes of the Ministry of Education in Xi'an Jiaotong University. The cells were cultured with the RPMI-1640 culture medium containing 10% FBS, supplemented with 100 U/ml penicillin and 100 *μ*g/ml streptomycin, in a 37°C, 5% CO_2_ incubator. Cells in the logarithmic growth phase were used in the following experiments.

### 2.2. The siRNA Synthesis and Transfection

The small interfering RNA (siRNA) for NEIL1 gene silencing was synthesized by GenePharma (Shanghai, China). The siNC was used as a negative control. The primer sequences are shown in Table 1. For transfection, the HCT116 and SW480 cells were cultured for 24 h, and the siRNA transfection was performed with the Polyplus transfection method, according to the manufacturer's instructions.

### 2.3. Plasmid Construction and Transfection

The pre-miR-7 expression vector was constructed by amplifying miR-7 with synthetic oligonucleotides and cloning it between the EcoRI and HindIII sites of the pcDNA6.2-GW/EmGFP vector (Invitrogen, Carlsbad, CA, USA). The putative miR-7 binding site in the 3′-UTR of NEIL1 was used to construct a wild-type or mutant reporter duplex. They were chemically synthesized and cloned into the pmirGLO vector between the SacI and XhoI sites, according to the manufacturer's instructions (Dual-Luciferase Reporter Assay System, Promega, USA). The HCT116 and SW480 cells were subjected to the transient transfection, and the transfected cells were cultured with the antibiotic-free RPMI-1640 medium for 24 h.

### 2.4. Bioinformatics Analysis

Data of the NEIL1 expression in CRC tissues were extracted from the Cancer Genome Atlas (TCGA) database (https://xenabrowser.net), and the Mantel-Cox analysis was performed. For the prediction of target miRNA, two common public databases were used, including the TargetScan (http://www.targetscan.org/vert_71) and miRDB (http://mirdb.org/miRDB) databases. Moreover, the online crosschecking tool (http://bioinfogp.cnb.csic.es/tools/venny/index.html) was used to identify the 3′-UTR of NEIL1 targeted by miR-7-5p. Furthermore, the Pearson correlation coefficient was used to analyze the relationship between the expression levels of miR-7-5p and NEIL1 in the CRC tissues.

### 2.5. MTT Analysis

The HCT116 and SW480 cells were seeded onto the 96-well plates at the density of 2500 cells/well. After 24 h, 48 h, and 72 h, the cell viability was analyzed using the MTT assay. Total of 20 *μ*l MTT was added to each well, and the plate was incubated at 37°C for 4 h. Thereafter, the supernatant was discarded, and 150 *μ*l DMSO was added into each well. Absorbance at 492 nm was measured using a microplate reader.

### 2.6. Apoptosis Assessment

At 48 h after transfection, the HCT116 and SW480 cells were harvested. The cells were washed and stained using the Annexin V-FITC/PI Apoptosis Detection Kit, according to the manufacturer's instructions. Flow cytometry was performed, and the level of apoptotic cells was quantified.

### 2.7. Quantitative Real-Time PCR

Total RNA was extracted from human CRC cells with TRIzol (Invitrogen). The cDNA was obtained with the StarScript II First-Strand cDNA Synthesis Mix Kit (GenStar, Beijing, China). Quantitative real-time PCR was performed with the RealStar SYBR Green qPCR Power Mixture (GenStar) on the IQ5 Multicolor qRT-PCR machine. The primers were synthesized by the Tsingke Biotech Co., Ltd., Beijing, China, and the primer sequences are shown in Table 1. The 20 *μ*l reaction system consisted of 10 *μ*l SYBR Green, 1 *μ*l primer each, 2 *μ*l cDNA, and 6 *μ*l ddH_2_O. Reaction conditions were set as follows: 95°C for 10 min, 95°C for 15 s, and 60°C for 1 min, for a total of 40 cycles, and 94°C for 1 min and 60°C for 3 min. The target gene expression levels were calculated with the 2^-*ΔΔ*Ct^ method. GAPDH and U6 were used as internal control.

### 2.8. Western Blot Analysis

The cells were lysed with RIPA buffer supplemented with protease inhibitors. Equal amounts of protein sample were separated by 10% SDS-PAGE, which was then transferred onto the PVDF membrane. After blocking with 5% nonfat milk at room temperature for 1 h, the membrane was incubated with the primary antibody (1 : 1000 dilution; Proteintech, Wuhan, Hubei, China) at 4°C overnight. Then, the membrane was incubated with the corresponding goat anti-rabbit secondary antibody (1 : 5000 dilution; Santa Cruz Biotechnology, Santa Cruz, CA, USA) at room temperature for 1 h. Thereafter, the color development was performed with the ECL method. The luminescent signal was detected and recorded by the Syngene G:Box system. GAPDH was used as the internal reference.

### 2.9. Statistical Analysis

Data were expressed as mean ± SD. The SPSS software was used for statistical analysis. The *t*-test was performed for the comparison between two independent groups. *P* < 0.05 was considered statistically significant.

## 3. Results

### 3.1. NEIL1 Inhibits Apoptosis and Increases Cell Viability of Human CRC Cells

**Figure 1:**
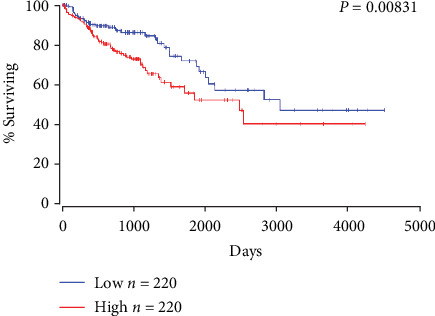
Survival prediction in cancer patients concerning NEIL1. For colorectal cancer cases, patients with low expression of NEIL1 had higher survival rates than patients with high expression of NEIL1.

**Figure 2:**
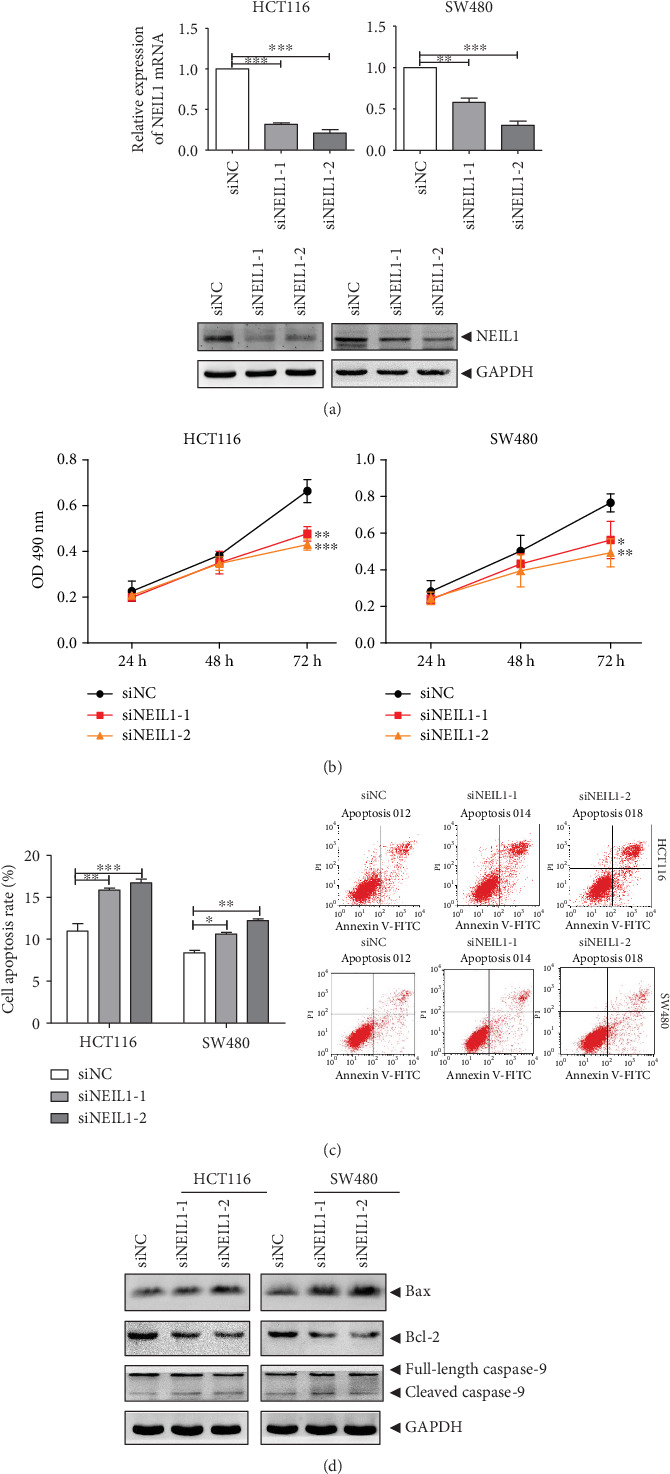
Inhibition of NEIL1 promotes apoptosis and inhibits cell viability in human colorectal cancer cells. HCT116/SW480 cells were treated with siNEIL1. (a) The mRNA and protein expression levels of NEIL1 were detected with the quantitative real-time PCR and Western blot analysis. (b) The cell viability of HCT116/SW480 cells treated with siNEIL1 was assessed with the MTT analysis. (c) At 48 h after transfection, the expression levels of apoptosis-regulated proteins in HCT116/SW480 cells were measured. (d) At 48 h after transfection, the apoptosis of siNEIL1-treated HCT116/SW480 cells was detected by flow cytometry. ^∗^*P* < 0.05, ^∗∗^*P* < 0.01, and ^∗∗∗^*P* < 0.001.

Data of the NEIL1 expression in the CRC tissues were extracted from the TCGA database, and the Mantel-Cox analysis revealed that patients with high expression of NEIL1 were associated with poor survival (Figure 1). Accordingly, two siRNAs targeting NEIL1 (siNEIL1-1 and siNEIL1-2) were designed and synthesized. These siRNAs and siNC were transfected into the HCT116 and SW480 human CRC cells, and the real-time quantitative PCR and Western blot were performed to detect the mRNA and protein expression levels of NEIL1. Our results showed that both the mRNA and protein expression levels of NEIL1 were significantly downregulated in the HCT116 and SW480 cells transfected with siNEIL1 (Figure 2(a)). Moreover, the cell viability was assessed with the MTT assay. Our results showed that, along with the downregulation of NEIL1 expression, the cell viabilities significantly declined in the transfected HCT116 and SW480 cells (Figure 2(b)). Detection of the cellular apoptosis with flow cytometry showed that, in the cells with downregulated NEIL1 expression levels, the apoptotic cells at early and late stages were both increased (Figure 2(c)). In addition, our results from the Western blot analysis showed that after the transfection of siNEIL1 in the human CRC cells, the Bax expression levels were significantly increased while the Bcl-2 expression levels were significantly decreased, resulting in the Bax/Bcl-2 balance moving towards apoptosis (Figure 2(d)). Moreover, the contents of cleaved and activated caspase-9 were also increased (Figure 2(d)). Taken together, these results suggest that reducing the NEIL1 expression in human CRC cells can promote cellular apoptosis and reduce the cell viability.

**Figure 3:**
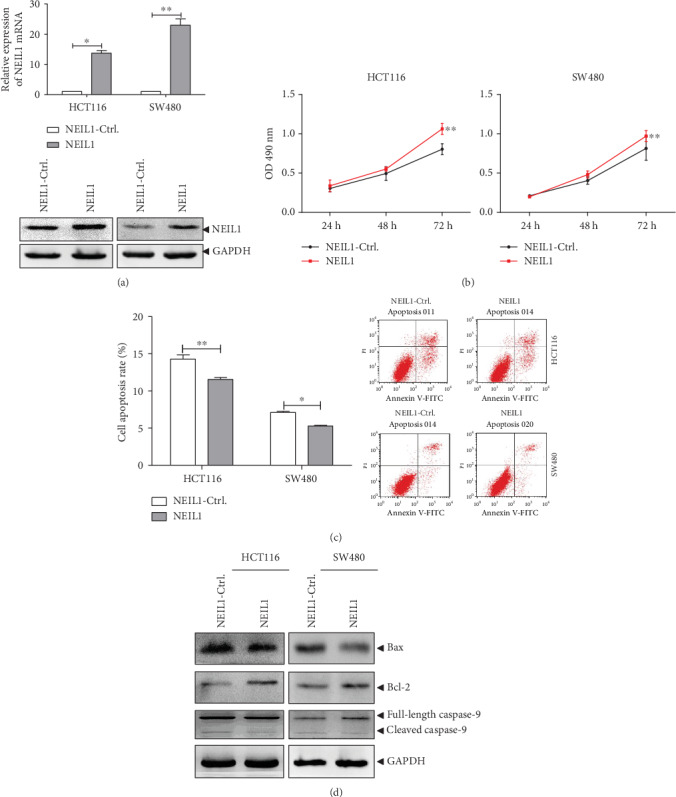
Overexpression of NEIL1 reduced apoptosis and increased cell viability in human colorectal cancer cells. NEIL1 overexpression plasmid was constructed and transiently transfected into HCT116/SW480 cells. (a) The mRNA and protein expression levels of NEIL1 were detected with the quantitative real-time PCR and Western blot analysis. (b) The cell viability of HCT116/SW480 cells overexpressed with siNEIL1 was assessed with the MTT analysis. (c) At 48 h after transfection, the apoptosis of siNEIL1-treated HCT116/SW480 cells was detected by flow cytometry. (d) At 48 h after transfection, the expression levels of apoptosis-regulated proteins in HCT116/SW480 cells were measured. ^∗^*P* < 0.05 and ^∗∗^*P* < 0.01.

To further confirm the role and effect of NEIL1, the recombinant plasmid with high expression of NEIL1 was constructed, which was transfected into the HCT116 and SW480 cells. Our results from the quantitative real-time PCR and Western blot analysis showed that the mRNA and protein expression levels of NEIL1 were upregulated in these transfected cells (Figure 3(a)). Moreover, our results from the MTT analysis showed that the high NEIL1 expression promoted the cell viability compared with the control group (Figure 3(b)). Furthermore, our results from the flow cytometry revealed that the high NEIL1 expression reduced the apoptotic cells (Figure 3(c)) and moved the Bax/Bcl-2 balance to become antiapoptotic. The contents of cleaved and activated caspase-9 were also decreased (Figure 3(d)). These findings were all contrary to the results obtained according to the NEIL1 expression downregulation. Taken together, our results showed that NEIL1 can inhibit the cellular apoptosis and increase the cell viability in human CRC cells, which might contribute to explaining the fact that the high NEIL1 expression in the CRC patients is associated with poor survival.

### 3.2. NEIL1's Negative Targeting Is Regulated by miR-7-5p

**Figure 4:**
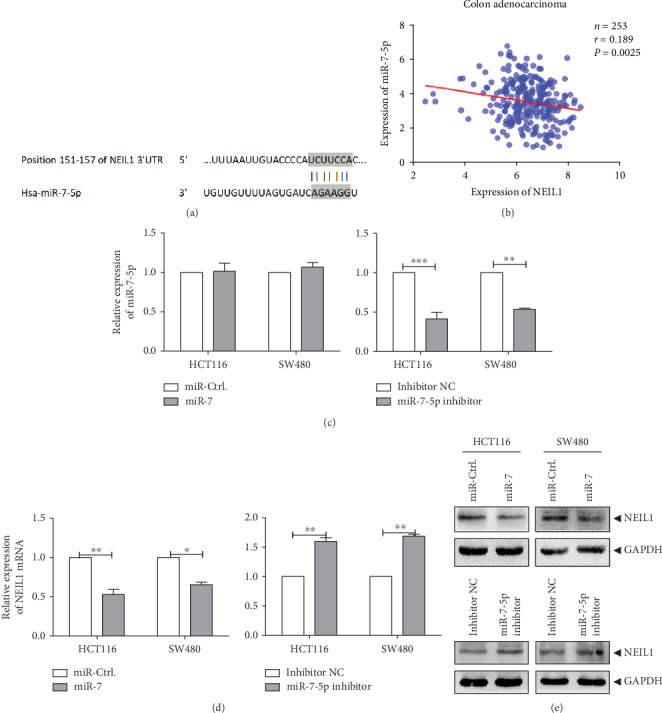
NEIL1 is negatively regulated by miR-7-5p. (a) Potential binding site of miR-7-5p in the NEIL1 3′-UTR. (b) The expression levels of miR-7-5p and NEIL1 were negatively correlated in colorectal cancer tissues and human colorectal cancer cells. (c) The miR-7-5p levels were measured by quantitative real-time PCR after high expression/inhibition of miR-7-5p. (d) The NEIL1 mRNA expression levels were measured by quantitative real-time PCR after high expression/inhibition of miR-7-5p. (e) The NEIL1 protein expression levels were detected with Western blot analysis after high expression/inhibition of miR-7-5p. ^∗^*P* < 0.05 and ^∗∗∗^*P* < 0.001.

MicroRNAs can negatively regulate the expression of target genes after transcription by binding to the gene's 3′-UTRs. The NEIL1 3′-UTRs targeted by miR-7-5p were found based on the TargetScan (http://www.targetscan.org/vert_71) and miRDB (http://mirdb.org/miRDB) common databases, in combination with the online crosschecking tool (http://bioinfogp.cnb. Csic.es/tools/venny/index.html) (Figure 4(a)). Moreover, our results showed that the expression levels of miR-7-5p and NEIL1 were negatively correlated, in the CRC tissues (Figure 4(b)) and human CRC cells (supplementary Fig. 1). Therefore, a recombinant plasmid highly expressing miR-7 was constructed, and the miR-7-5p inhibitor was designed and synthesized (Table 1). The miR-7-high expression plasmid and the control plasmid were transfected into HCT116 and SW480 cells, respectively. Our results from the real-time quantitative PCR showed that there was no significant difference in miR-7-5p between cells transfected with miR-7-5p and control cells (Figure 4(c)). Moreover, the miR-7-5p inhibitor and inhibitor-NC controls were transfected into the HCT116 and SW480 cells, respectively. As shown in Figure 4(c), in these transfected cells, the expression level of miR-7-5p was significantly downregulated in cells transfected with the miR-7-5p inhibitor (Figure 4(c)). Importantly, our results from the real-time quantitative PCR and Western blot results showed that the high expression levels of miR-7 significantly decreased the mRNA and protein expression levels of NEIL1 in the HCT116 and SW480 cells compared with the control group, while the transfection with the miR-7-5p-inhibitor significantly upregulates the mRNA and protein expression levels of NEIL1 in these cells (Figures 4(d) and 4(e)). These results indicate that the NEIL1 expression level is negatively regulated by the miR-7-5p transcription in the human CRC cells.

### 3.3. miR-7-5p Negatively Regulates NEIL1 in Human CRC Cells, Inhibits Cellular Apoptosis, and Promotes Cell Viability

**Figure 5:**
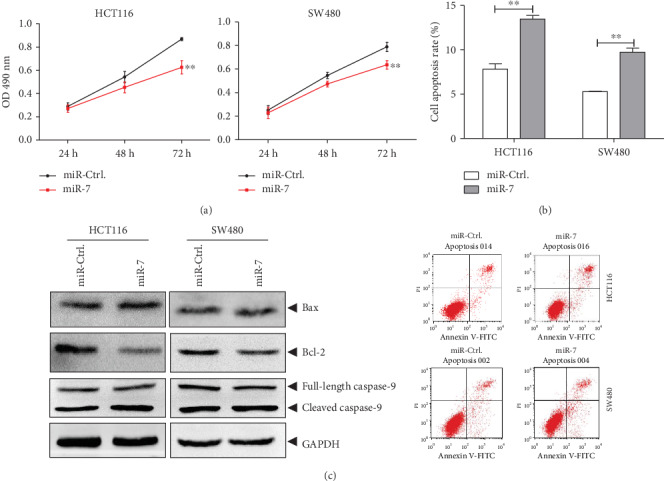
Overexpression of miR-7-5p promotes apoptosis and inhibits cell viability in human colorectal cancer cells. HCT116/SW480 cells were transfected with miR-7-5p overexpression plasmid and negative control (miR-Ctrl.). (a) At 24 h, 48 h, and 72 h after transfection, the survival rates of HCT116/SW480 cells were determined with the MTT assay. (b) Apoptosis of HCT116/SW480 cells was measured by flow cytometry at 48 h after transfection. (c) Expression levels of apoptosis-regulated proteins in HCT116/SW480 cells were measured at 48 h after transfection. ^∗∗^*P* < 0.01.

**Figure 6:**
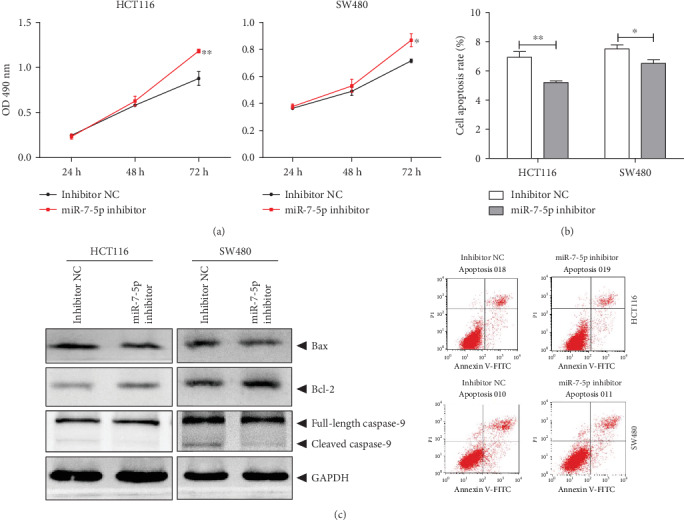
Inhibition of miR-7-5p reduces apoptosis and increases cell viability in human colorectal cancer cells. HCT116/SW480 cells were transfected with miR-7-5p inhibitor and negative control (NC) inhibitor. (a) At 24 h, 48 h, and 72 h after transfection, the cell viabilities of HCT116/SW480 cells were detected with the MTT assay. (b) Apoptosis of HCT116/SW480 cells was measured by flow cytometry at 48 h after transfection. (c) Expression levels of apoptosis-regulated proteins in the HCT116/SW480 cells were measured at 48 h after transfection. ^∗^*P* < 0.05 and ^∗∗^*P* < 0.01.

The role of miR-7-5p in the human CRC cells was further investigated. Our results from the MTT assay showed that the high expression of miR-7 significantly decreased the viabilities of HCT116 and SW480 cells, compared with the control group (Figure 5(a)). Moreover, our results from the flow cytometry showed that the high expression of miR-7 increased the number of apoptotic cells at the early and late stages (Figure 5(b)), and the Bax/Bcl-2 balance moved towards apoptosis. The contents of cleaved and activated caspase-9 were elevated (Figure 5(c)). These results suggest that the high expression of miR-7 in the human CRC cells would promote the cellular apoptosis and reduce the cell viability, consistent with results from the siNEIL1 transfection. In contrast, compared with the control group, transfection of the miR-7-5p-inhibitor promoted the viabilities of HCT116 and SW480 cells (Figure 6(a)). Moreover, the apoptotic cells were decreased (Figure 6(b)), and the Bax/Bcl-2 balance moved to be antiapoptotic. Furthermore, the contents of the cleaved and the activated caspase-9 declined (Figure 6(c)), consistent with the results from the experiments concerning the high expression of NEIL1.

**Figure 7:**
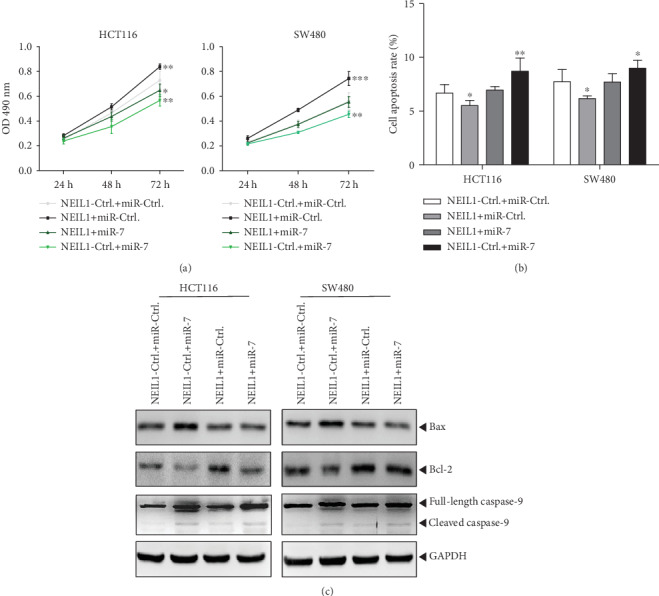
miR-7 overexpression reverses the effect of NEIL1 overexpression. NEIL1 overexpression plasmid or its control and miR-7 overexpression plasmid or its negative control (miR-Ctrl.) were cotransfected into the HCT116/SW480 cells. (a) At 24 h, 48 h, and 72 h after transfection, MTT assay was used to assess the cell proliferation ability of each group on HCT116/SW480 cells. (b) Apoptosis of HCT116/SW480 cells was measured by flow cytometry at 48 h after transfection. (c) Expression of apoptotic regulatory proteins in HCT116/SW480 cells was measured at 48 h after transfection. ^∗^*P* < 0.05 and ^∗∗^*P* < 0.01.

The above results suggest that, in the human CRC cells, miR-7-5p can promote the cellular apoptosis and inhibit the cell viability, opposing the effects of NEIL1. Therefore, miR-7-5p can negatively regulate NEIL1 in the human CRC cells, exerting the effects on cellular apoptosis and cell viability. Next, the NEIL1 and miR-7 were expressed in the HCT116 and SW480 cells, both respectively and simultaneously. Our results showed that the miR-7 overexpression significantly reduced the NEIL1-enhanced cell viability (Figure 7(a)) and NEIL1-induced apoptosis inhibition (Figures 7(b) and 7(c)) in the human CRCs. Taken together, these results indicate that in the human CRC cells, changes in the expression of miR-7 can negatively regulate NEIL1, subsequently affecting cellular apoptosis and cell viability.

## 4. Discussion

DNA base excision repairing (BER) system is responsible for detecting and repairing the DNA damage caused by alkylation, oxidation, cyclization, single-strand DNA fragmentation, and deamination, which is a very active DNA repairing method in the eukaryotic cells. The BER system includes the short- and long-patch BER processes, mainly involving the following basic processes: identification and removal of damaged bases by DNA transglucosylase, forming an intermediate containing a purine-free pyrimidine-free site (AP); in AP, endonuclease 1 cleaves the 5′-end of the phosphodiester backbone, till the AP site; removal of the 5-glycosyl fragment; synthesis of a new base by the DNA polymerase such as DNA polymerase *β*; and successful repair of the gap by DNA ligase I or III and X crosscomplementing protein 1.

CRC is the third most common cancer in the world and ranks the second in terms of global cancer mortality. In recent years, the morbidity and mortality rates of CRC cases in Asian countries have increased rapidly. The development of CRC is a multipathway process, involving the regulation of tumor suppressor genes and oncogenes. To achieve better treatment, it is of great significance to identify the new diagnostic biomarkers and to develop the molecular-regulating mechanisms during the CRC pathogenesis and development.

As the major regulator of BER, NEIL1 is associated with the development of many types of cancers. In this study, we aimed to determine the role of NEIL1 in CRC and the related mechanisms. CRC patients with high expression of NEIL1 always survived poorly, indicating that NEIL1 may play an important role in the development of CRC. Our results indicated that the downregulation of NEIL1 significantly inhibited the proliferation of CRC cells and slowed the cancer development. NEIL1 inhibited the CRC cells by upregulating the antiapoptotic gene Bcl-2 and downregulating the proapoptotic genes (i.e., Bax and caspase-9). The Bcl-2 family is a family of apoptosis-related genes, which can be divided into the anti- and proapoptotic genes. After inactivation of Bcl-2, apoptosis can be inhibited by forming a heterodimer with Bax. There is a balance between Bcl-2 and Bax. Increased Bax would promote apoptosis, while excessive Bcl-2 inhibited cellular apoptosis. The proportion of Bax/Bcl-2 during the apoptotic process plays a key role in cancer progression. The caspase family acts as executors of apoptosis, and caspase-9, a key enzyme in the apoptotic pathway, could activate the poly (ADP-ribose) polymerase, leading to subsequent apoptotic events in the downstream cascade. It has been well known that apoptosis is the result of a series of highly regulated caspase cascades, in which caspase-9 plays a key role. Activated caspase-9 can cleave DNA and inactivate the related proteases in the DNA damage repairing system, leading to apoptotic processes. Taken together, our results demonstrate that NEIL1 inhibits the CRC apoptosis by inhibiting the caspase-9 signaling pathway.

It has been demonstrated that miRNAs target the 3′-UTR of target genes to inhibit the gene expression and affect its biological function. Discovering specific miRNAs and their target genes associated with cancer development will provide important clues for the disease diagnosis and treatment. In our previous study, the bioinformatics found that NEIL1 was a negative target for miR-7-5p in CRC cells. miR-7-5p usually plays a tumor suppressor role in bladder cancer, breast cancer, CRC, and glioblastoma. Moreover, it has been shown that miR-7 can decrease the cyclin D1 expression and increase the p21, caspase-3, and Bax expressions, inhibiting the CRC cell proliferation and inducing apoptosis [13]. Furthermore, a previous study has shown that miR-7 targets the oncogenic YY1 transcription factor in CRC, ultimately leading to inhibition of p53 [13]. In line with these findings, our results showed that miR-7 acted as a tumor suppressor, especially in CRC. Recently, DNA hypermethylation is a major epigenetic modification that occurs on the CpG islands, associated with abnormal miRNA expression in cancers [14]. To date, however, the related roles of miR-7-5p and NEIL1 have not been reported in CRC. For the first time, we found that miR-7-5p expression was negatively correlated with NEIL1 in CRC tissues. Furthermore, our results indicated that overexpression of miR-7-5p reversed the biological processes induced by high NEIL1 expression in CRC cells. miR-7 may serve as a biomarker or therapeutic target gene in CRC, as well as in the precancerous lesions (inflammatory responses).

In conclusion, the expression of NEIL1 is inversely related to the survival of patients with CRC. It is regulated by miR-7-5p. It promotes cell viability by inhibiting apoptosis of human colon cancer cells. These findings provide new insights into the regulatory network of tumor cell viability changes regulated by NEIL1 and provide evidence for NEIL1 as a tumor promoter in CRC pathology and a potential therapeutic target for CRC therapy.

## Figures and Tables

**Table 1:** Primers and oligonucleotides used in this study.

Primer	Sequence (5′-3′)
siNC-sense	UUCUCCGAACGUGUCACGUTT
siNC-antisense	ACGUGACACGUUCGGAGAATT
siNEIL1-1-sense	GGGCGCAAGUCCCGCAAAA
siNEIL1-1-antisense	UUUUGCGGGACUUGCGCCC
siNEIL1-2-sense	GCUCCCACAGUGCCCAAGA
siNEIL1-2-antisense	UCUUGGGCACUGUGGGAGC
NEIL1-forward	AAGTCAGGTTCTTCCGCCAC
NEIL1-reverse	CGGTAGGCACTGCTCTCAAAG
GAPDH-forward	GGAGAGTTTCGGTTTAGTTTGGA
GAPDH-reverse	ACCTTTGTTGCCTATTTGCAGT
Pre-miR-7-sense	AATTCAGATTAGAGTGGCTGTGGTCTAGTGCTGTGTGGAAGACTAGTGATTTTGTTGTTCTGATGTACTACGACAACAAGTCACAGCCGGCCTCATAGCGCAGACTCCCTTCGACA
Pre-miR-7-antisense	AGCTTGTCGAAGGGAGTCTGCGCTATGAGGCCGGCTGTGACTTGTTGTCGTAGTACATCAGAACAACAAAATCACTAGTCTTCCACACAGCACTAGACCACAGCCACTCTAATCTG
miR-7-5p RT	GTCGTATCCAGTGCGTGTCGTGGAGTCGGCAATTGCAC TGGATACGACCTAGTGG
miR-7-5p-forward	ACGUGACACGUUCGGAGAATT
miR-7-5p-reverse	GCCAAACCUCAGUGAAUUUTT
U6-RT	AAAUUCACUGAGGUUUGGCTT
U6-forward	CGCAGAGGUACUGCAUAUATT
U6-reverse	UAUAUGCAGUACCUCUGCGTT

## Data Availability

The data used to support the findings of this study are available from the corresponding author upon request.

## Abbreviations

CRC: Human colorectal cancer
NEIL1: Nei endonuclease VIII-like1
miRNAs: MicroRNAs
3′-UTR: 3′-Untranslated region.

## Supplementary Materials

Supplementary Materials Suppl. Fig. 1: quantitative real-time PCR was used to detect the mRNA expression levels of NEIL1 and miR-7-5p in human colon cancer cells.
