# Supplementary material for: Nei Endonuclease VIII-Like1 (NEIL1) Inhibits Apoptosis of Human Colorectal Cancer Cells
Source: Biomed Res Int. 2020 Jun 26;2020:5053975. doi: 10.1155/2020/5053975 (PMC7336199; doi:10.1155/2020/5053975)

Nei endounuclease VIII-like1 (NEIL1) inhibits apoptosis of human colorectal cancer cells

Wanjuan Xue[1,#], Yongcheng Liu[2,#], Ningning Xin[3,#], Jiyu Miao[4], Juan Du[1], Yu Wang[1], Haiyan Shi[1], Yameng Wei[1], Huahua Zhang[1], Yani Chen[1], Yi Gao[1], Dan Li[1], Yun Feng[1], Jing Yan[1], Jing Zhang[1], Ni Hou[4], Chen Huang[4,*], Jiming Han[1,*]

[1]Department of Cell Biology and Genetics, Medical College of Yan'an University, Yan'an 716000, Shannxi, China;

[2]Department of Pathology, Yan'an University Affiliated Hospital, Yan'an 716000, Shannxi, China;

[3]School infirmary, XianYang Normal University, Xianyang 712000, Shannxi, China;

[4]Department of Cell Biology and Genetics, School of Basic Medical Sciences, Xi'an Jiaotong University Health Science Center, Xi'an 710061, Shannxi, China.

[#]These authors contributed equally to this work.

Corresponding authors:

Chen Huang, Department of Cell Biology and Genetics, School of Basic Medical Sciences, Xi'an Jiaotong University Health Science Center, No. 76, Yanta Road, Xi'an 710061, Shaanxi, China. Tel: 86-029 8265 7723. E-mail: hchen@mail.xjtu.edu.cn

Jiming Han, Department of Clinical Medicine, Medical College of Yan'an University, No. 38, Guanghua Road, Yan'an 716000, Shaanxi, China. Tel: 86-911 2412 444. Email: yadxsci@163.com

Running title: Effects of NEIL1 on human CRC

Abbreviations: CRC = human colorectal cancer; NEIL1 = Nei endounuclease VIII-like1; miRNAs = microRNAs; 3'-UTR = 3'-untranslated region

Suppl Figure 1

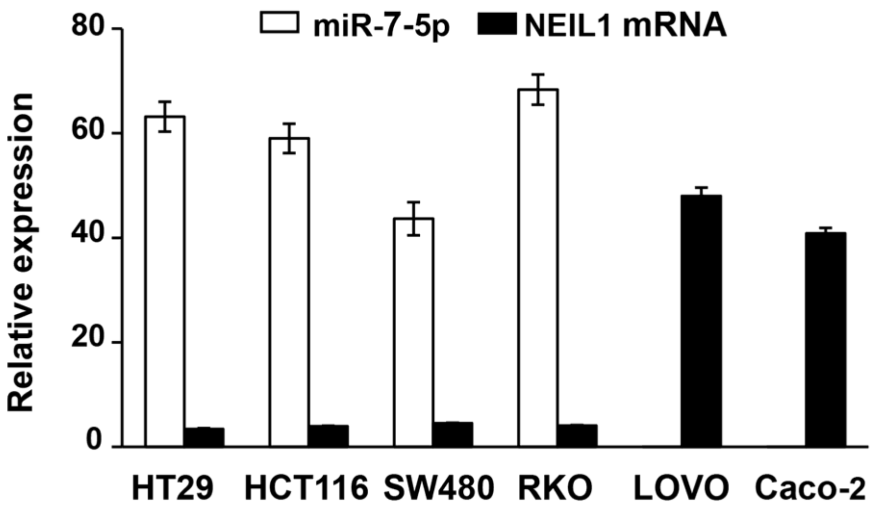

Supplement: Supplementary Materials — Suppl. Fig. 1: quantitative real-time PCR was used to detect the mRNA expression levels of NEIL1 and miR-7-5p in human colon cancer cells. [file 5053975.f1.pdf]
